# Platelet membrane-coated nanoparticles: Bioengineering principles, quality control, and translational opportunities

**DOI:** 10.1063/5.0315665

**Published:** 2026-05-22

**Authors:** Ncobile Bagezile Mdlovu, Liling Delila, Si-Han Wu, Thierry Burnouf

**Affiliations:** 1 International Ph.D. Program in Biomedical Engineering, College of Biomedical Engineering, Taipei Medical University, Taipei, Taiwan; 2 Graduate Institute of Biomedical Materials and Tissue Engineering, College of Biomedical Engineering, Taipei Medical University, Taipei, Taiwan; 3 Graduate Institute of Nanomedicine and Biomedical Engineering, College of Biomedical Engineering, Taipei Medical University, Taipei, Taiwan; 4 International PhD Program in Cell Therapy and Regeneration Medicine, Taipei Medical University, Taipei, Taiwan

## Abstract

Platelet membranes (PMs) are increasingly explored as bioinspired coatings for nanoparticles (NPs), providing improved immune evasion, prolonged circulation, and disease-homing properties that enhance targeted drug delivery. Unlike conventional NPs that rely mainly on passive targeting, PM-coated systems expose platelet surface markers such as CD47, GPIb, and P-selectin, enabling vascular adhesion and selective localization to tumors or thrombi. PM–NPs are thus increasingly regarded as promising carriers for oncology, cardiovascular, and infectious disease therapies. This review introduces the various bioengineering principles underlying PM–NP fabrication, including points to consider for platelet sourcing, membrane isolation, and coating strategies. Achieving reliable quality control (QC) and reproducibility depends on rigorous assessment of critical formulation variables, including nanoparticle size, surface charge, and the preservation of functional membrane proteins. The implementation of scientific approaches and regulatory standardization frameworks, such as the Minimal Information for Studies of Extracellular Vesicles guidelines, and Food and Drug Administration/European Medicines Agency (FDA/EMA) regulatory expectations, is critical to establish reproducibility and facilitate regulatory acceptance of PM–NP technologies, guiding their advancement toward clinical-grade production. Furthermore, we highlight translational opportunities and the complementary potential of platelet-derived extracellular vesicles, which share similar surface markers, yet offer intrinsic nanoscale size, endogenous bioactivity, and improved stability. By integrating robust engineering design with standardized QC practices, PM–NPs can progress from laboratory research to clinically viable therapeutics, establishing a relevant benchmark for future cell membrane-based nanomedicines.

## I. INTRODUCTION

Effective therapeutic delivery remains a major challenge in nanomedicine.^1^ Conventional nanoparticles, despite their structural tunability and high drug-loading potential, are rapidly cleared from the bloodstream, limiting their accumulation in target tissues. Most rely on passive targeting through the enhanced permeability and retention (EPR) effect, yet only a very small fraction, typically less than 1%, actually reaches solid tumors.^2^ The majority are rapidly cleared by the reticuloendothelial system (RES), leading to low efficacy and unwanted systemic exposure. Despite over 40 000 studies on active targeting in the last few years, clinical outcomes remain disappointing.^3–6^ The lack of effective targeting results in nonspecific distribution, systemic toxicity, and adverse side effects. PEGylation was one of the first strategies introduced to reduce RES clearance and improve NP circulation.^7,8^ Yet, its clinical translation has been limited by anti-PEG antibody formation, accelerated blood clearance (ABC), and the inconsistency of the EPR effect in dense tumors, such as gliomas.^3,9^ These limitations have prompted the development of a biomimetic drug nanocarrier platform, which combines the customizable properties of synthetic NPs with cell-derived membranes to enhance selective targeting, stability, and biocompatibility.^10^

The field of cell membrane-coated nanoparticles (CMNPs) has expanded rapidly. Different cell types are now used as membrane sources depending on the disease being targeted. Red blood cell (RBC) membranes were the first to be used because they have a prolonged circulation time and help avoid immune clearance. Leukocyte membranes (LM) have been studied for targeting inflammation, and cancer cell membranes (CCM) for targeting tumors of the same type. More recently, stem cell-derived membranes have also been investigated because of their regenerative potential and ability to move toward tumors.^8,11^ However, despite this progress, the lack of standard methods for membrane isolation, coating, and quality control (QC) remains a major bottleneck for reproducibility and clinical translation. In this context, platelet membranes (PMs) are a uniquely versatile platform, combining the long-circulation advantage of RBCs with the active targeting and immune-modulatory capabilities of leukocytes.

PMs are attracting special interest, due to their ready accessibility from clinical-grade allogeneic human platelet concentrates (PCs) or other sources such as autologous platelet-rich plasma (PRP), inherent low immunogenicity, prolonged circulation, and natural disease-homing capacity.^10,12^ PMs express CD47, which enables immune evasion and prolonged circulation,^13,14^ while glycoproteins such as GPIb and P-selectin mediate adhesion to collagen and tumor-associated proteins. Upon platelet activation, P-selectin is exposed on the platelet surface, enabling binding to P-selectin glycoprotein ligand-1 (PSGL-1) or CD44, both highly expressed on tumor cells.^15^ These interactions provide PM-coated nanoparticles (PM–NPs) with unique advantages as drug delivery systems (DDS).

Despite these merits, unresolved engineering and translational challenges remain in the applications of PM–NPs. Preparation of PMs is not standardized, while fabrication of NP cores varies across laboratories, affecting scalability and reproducibility. The coating procedures of membranes, e.g., via extrusion or sonication, remain poorly understood and result in incomplete coverage.^16,17^ To advance toward clinical translation, standardized protocols and stringent quality control (QC) are required, which should be applied not only to the final PM–NPs but also to each component of the DDS, the NP core, the therapeutic cargo, and the PMs, as well as the overall engineering workflow. Quantitative evaluation of membrane protein orientation has become an essential QC criterion for clinical translation. Moreover, appropriate storage conditions to preserve cargo stability and membrane integrity are critical for ensuring reproducibility, functional stability, and regulatory approval.^10,18^

From a broader perspective, PM–NPs can be positioned within the continuum of extracellular vesicle (EV)-based therapeutics outlined by the International Society for Extracellular Vesicles (ISEV), which encompasses native EVs, drug-loaded EVs, engineered EVs, and EV-mimetic nanovesicles.^19^ In this framework, PM–NPs can be broadly considered EV-mimetics, in which synthetic NP cores are cloaked with biologically derived lipid–protein membranes. In contrast, p-EV-coated nanoparticles and cell-derived artificial exosomes occupy an intermediate space between engineered EVs and EV mimetics. Conceptually embedding PM–NPs within this EV landscape enables the application of established guidelines, such as the Minimal Information for Studies of Extracellular Vesicles (MISEV) recommendations, and broader chemistry, manufacturing, and controls (CMC) principles to platelet-based nanocarriers.

While PMs and EVs have been mentioned in broader reviews of cell membrane-coated NPs,^12,20,21^ a focused analysis of their bioengineering design, QC, and translational potential is still lacking. Here, we address these gaps by highlighting the bioengineering of PM–NPs, with emphasis on design principles, QC, preclinical testing, and challenges of scaling preclinical success toward clinical applications.

## II. SOURCING OF PLATELET MEMBRANES

### A. PC as a scalable source of PMs

The first step in engineering PM–NPs is securing a reliable and clinically suitable source of PMs. While autologous PRP has been widely used in laboratory studies,^22–24^ its variability, lack of standardization, and inconvenience for some patients with limited venous access limit scalability and use. In contrast, PCs obtained from healthy donors and manufactured under good manufacturing practice (GMP) conditions for transfusion medicine represent a more consistent and clinically approved source of PMs.^21,25^ PCs can be obtained from whole-blood donations processed through buffy coat, or collected directly from a single donor by a dedicated apheresis technology, which typically yields 230–250 ml of platelet concentrate per procedure. Pooling donations from multiple donors is suggested to reduce batch-to-batch variability, thereby improving reproducibility and scalability for downstream applications, provided infectious risks are controlled.^25^ Despite this, their use as a source of PMs remains underexploited. Leveraging PCs could therefore facilitate scalability, reduce variability, and support regulatory approval of PM-based nanocarriers.

In comparison, platelets derived from induced pluripotent stem cells (iPSCs) are emerging as a potential alternative. They could provide, in principle, an unlimited source of platelets without relying on donors, and they offer the possibility for genetic modification, such as human leukocyte antigen (HLA) knockout, to allow universal compatibility.^26^ However, important challenges still limit their clinical translation. These include lower production yields than in natural platelet formation, difficulty in reproducing the full protein profile and function of primary platelets, and the high cost and regulatory complexity of engineered cell-derived products.^27^ Therefore, although iPSC technology is a promising future option for standardized and programmable membrane sources, primary PCs remain at present the most pragmatic and scalable source for the development of biomimetic nanocarriers.^25,28^

### B. Extraction and isolation of PMs

**FIG. 1.**
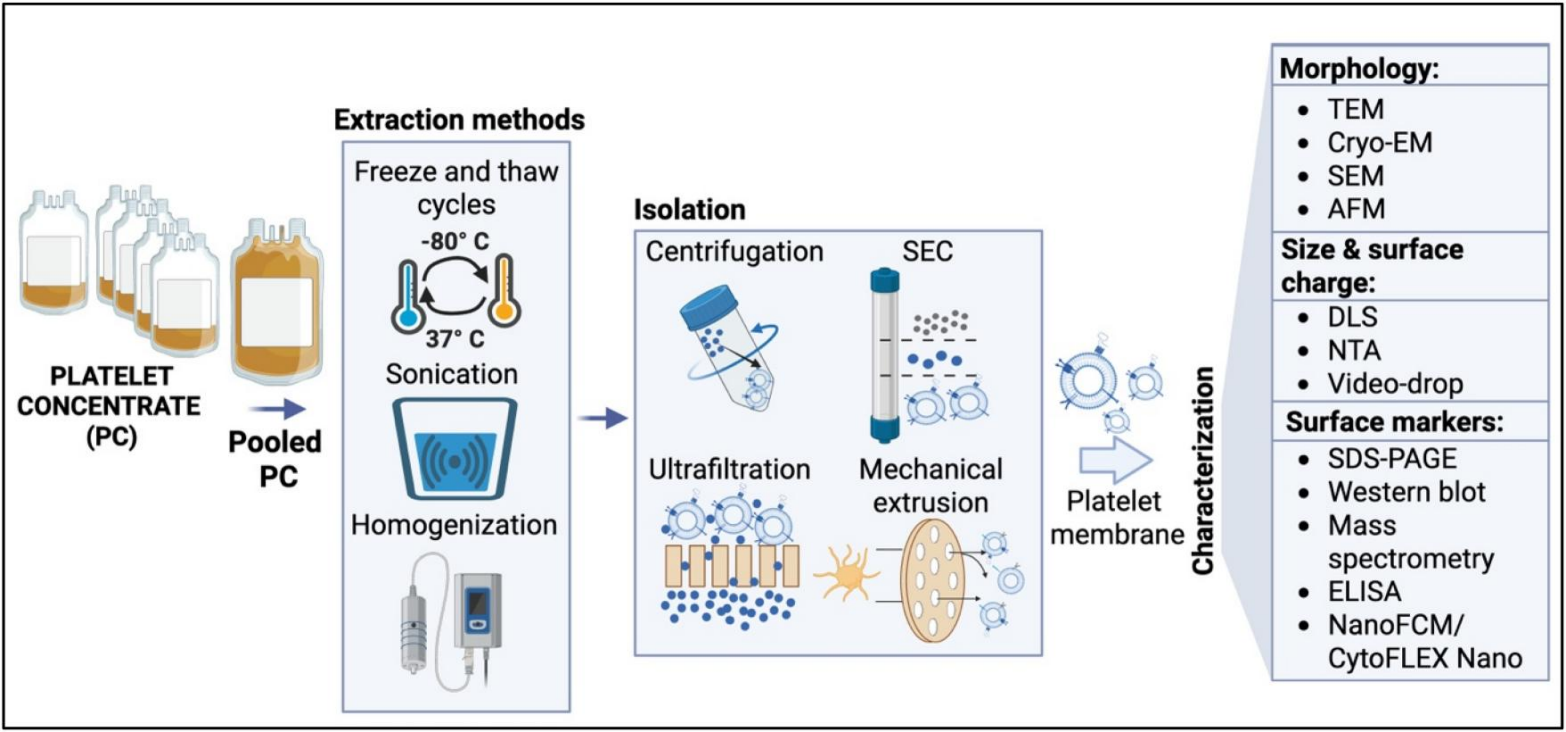
Extraction, isolation, and characterization of platelet membranes (PMs) derived from clinical-grade platelet concentrates (PCs). PCs obtained from healthy donors are pooled and subjected to physical disruption using freeze–thaw cycles (−80/+37 °C), sonication, and homogenization to rupture the platelet structure and release membranes. The resulting membranes are isolated through centrifugation, ultrafiltration, size-exclusion chromatography (SEC), and mechanical extrusion to yield nanosized platelet membrane vesicles. Characterization includes: (i) morphology analysis by transmission electron microscopy (TEM), cryo-electron microscopy (cryo-EM), scanning electron microscopy (SEM), and atomic force microscopy (AFM); (ii) particle size and surface charge determination using dynamic light scattering (DLS), nanoparticle tracking analysis (NTA), and video-drop measurement; and (iii) protein and surface marker profiling via sodium dodecyl sulfate-polyacrylamide gel electrophoresis (SDS-PAGE), Western blotting, mass spectrometry, enzyme-linked immunosorbent assay (ELISA), and nano-flow cytometry for single-vesicle analysis of platelet-specific markers (e.g., CD41, CD61, CD62P, and CD47) and discrimination between coated nanoparticles and free vesicles.

Several methods have been applied to isolate PMs from platelet sources, including repeated freeze–thaw cycles (−80/37 °C), homogenization, and sonication.^15,29^ Following membrane extraction, isolation techniques such as centrifugation, ultrafiltration, size-exclusion chromatography (SEC), and mechanical extrusion are employed to obtain the nanosized PM vesicles from PCs (Fig. 1). Each approach requires careful optimization to preserve membrane integrity and retain functional proteins important for targeting. However, technical variables, such as sonication frequency and duration, extrusion cycles, and freeze–thaw conditions, remain inconsistent among studies, leading to variable membrane quality and properties.^24,30,31^

## III. PRINCIPLES OF PLATELET-COATED NANOPARTICLES FOR DRUG DELIVERY

Coating NPs with PMs is a biomimetic strategy that combines synthetic cores with natural surface functionalization.^32^ The fabrication method used to wrap PMs around NPs is critical to product performance, as it must ensure uniform coverage, structural stability, and retention of functional proteins.^33^ Regardless of coating method, key PM proteins should remain preserved, including the immunomodulatory proteins CD47, CD55, and CD59, the integrins αIIbβ3, α2β1, α5β1, and α6β1, and receptors such as GPIb-IX-V complex, GPVI, CLEC-2, and CD36 (GPIV). Depending on the intended application, preservation of P-selectin may also be important for targeting.^34,35^ Yet, studies show that a large proportion (>90%) of NPs are only partially coated,^17^ underscoring the need for further optimization.

Two commonly applied approaches are extrusion and sonication. In extrusion, repeated passage of NPs and PMs through porous membranes promotes fusion, while in sonication, ultrasonic energy drives the wrapping process.^16^ Conventional extrusion uses repeated mechanical force and several processing cycles. This can introduce variability and may damage the membrane or lead to “Janus-like” partial coating. Although the method is robust and simple, it is labor-intensive at the bench scale and not very efficient. It typically takes about 30 min to prepare 2 ml and may cause a sample loss of 30%–50%. In contrast, microfluidic methods are emerging as an alternative, although they have been little studied for membrane coating of NPs. They provide a controlled laminar flow environment in which parameters such as flow rate ratio (FRR) and total flow rate (TFR) can be adjusted precisely to control mixing. Under these conditions, mixing occurs primarily by diffusion rather than turbulence, which allows better control of NP-membrane interactions and reduces stress on the membrane. This favors rapid and more uniform self-assembly of membrane-coated NPs. This technique has already been used in sensitive biological applications, such as DNA handling and cell encapsulation, where gentle processing is critical.^8,36,37^ Therefore, microfluidic strategies may improve coating uniformity, reduce the proportion of partly coated particles, enhance batch-to-batch reproducibility, and reduce material loss. This could help overcome an important barrier to scalable production.

Hybrid systems, in which PMs are combined with other membranes, enable multifunctional versatility of the coating.^38–40^ The chemistry of PMs also supports conjugation through abundant amine and thiol groups, enabling functionalization with biomolecules.^20^ These bioengineered systems exhibit enhanced circulation, improved pharmacokinetics, and selective tumor or thrombus targeting compared to uncoated NPs. An important factor influencing coating efficiency is the ratio of membrane to NP core. An insufficient number of PMs produces incomplete or “patchy” coatings, compromising stability, immune evasion, and targeting. Conversely, excessive PMs may waste biological material and promote aggregation. Balancing this ratio is essential to preserve activity and therapeutic outcome, yet current practice varies widely, with some groups reporting ratios by mass and others by particle number.^22,41^ Inconsistency in how membrane-to-core ratios are defined also complicates cross-study comparison. Harmonizing these definitions, potentially through EV-inspired identity and purity metrics, would facilitate reproducibility and eventual GMP alignment. Going forward, the field must emphasize: (a) controlled coating workflows that minimize partially coated particles; (b) quantitative QC assays to confirm surface protein retention and membrane uniformity; and (c) harmonized definitions of coating ratios, compliant with GMP requirements. These refinements will be essential to progress from proof-of-concept PM–NPs toward clinically viable biomimetic nanocarriers.

## IV. SELECTION AND ENGINEERING OF SYNTHETIC CORES FOR MEMBRANE COATING

**FIG. 2.**
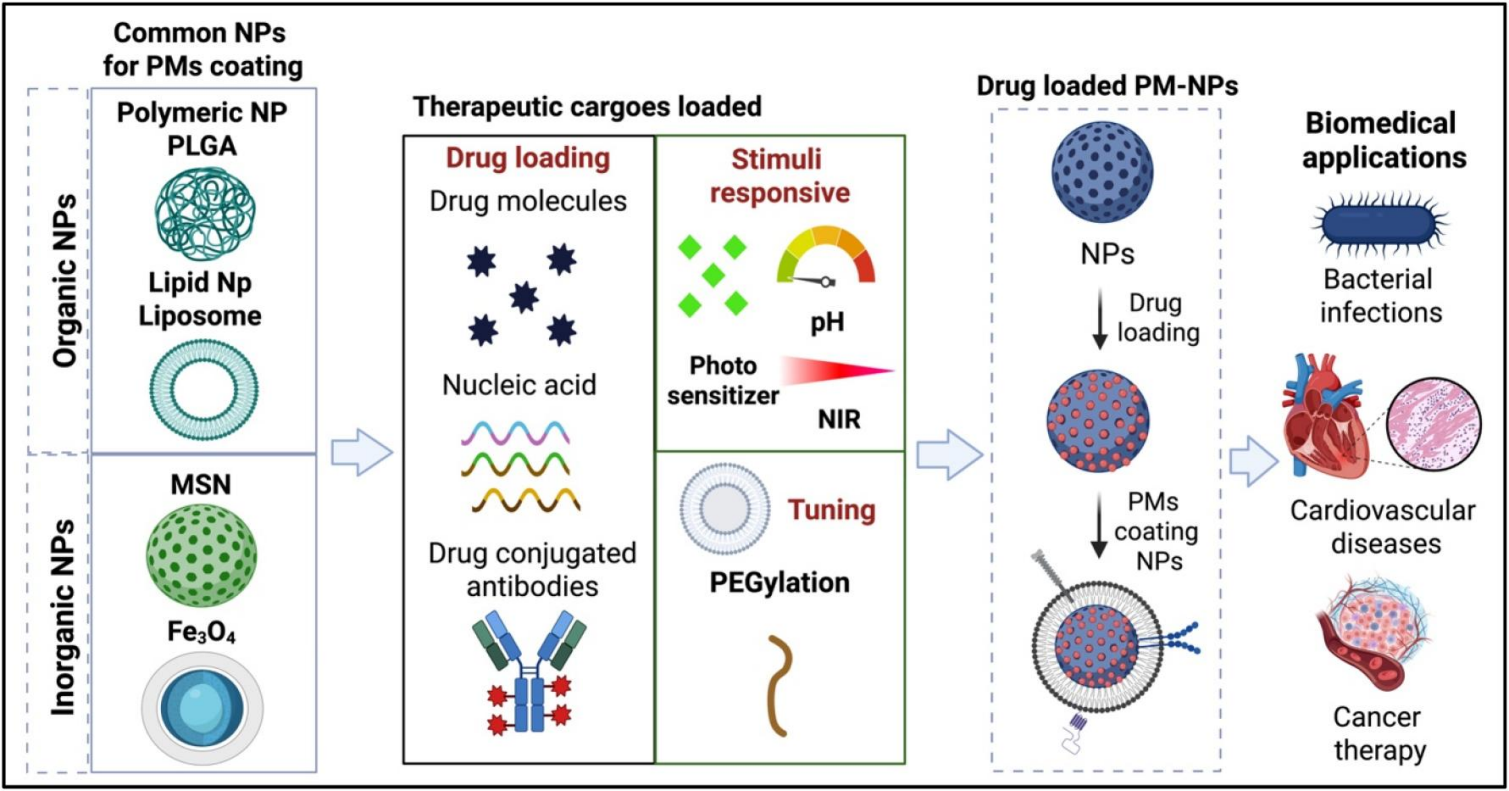
Schematic illustration of multifunctional nanoparticle-based drug delivery systems. Different types of nanoparticles (NPs), including polymeric nanoparticles (PLGA), lipid nanoparticles (liposomes), mesoporous silica nanoparticles (MSN), and iron oxide nanoparticles (Fe_3_O_4_), have been evaluated as platforms for therapeutic applications. These nanoparticles can be loaded with various therapeutic agents, such as small drug molecules, nucleic acids, and drug-conjugated antibodies. Functionalization enables stimuli-responsive release (e.g., triggered by pH, light/near-infrared (NIR) irradiation, or photosensitizers) and surface tuning (e.g., PEGylation for improved stability and circulation). The engineered nanoparticles are designed for targeted delivery to specific tissues or cells, including pathogenic bacteria, cardiac tissue, or tumor microenvironments, enhancing therapeutic efficacy and minimizing side effects.

The intended therapeutic application is a key determinant in selecting NP cores for biomimetic systems. Beyond the choice of core material, physicochemical parameters such as particle size, morphology, surface charge, and surface functionalization strongly influence payload capacity, circulation stability, and coating efficiency.^13^ Smaller cores are generally more efficiently wrapped by cell membranes, while PEGylation or the introduction of reactive surface groups can further reduce nonspecific protein adsorption, modulate drug release kinetics, and enhance membrane attachment.^42,43^ Synthetic cores are commonly fabricated from polymers, lipids, or inorganic compounds^44^ (Fig. 2). Among polymeric materials, poly (lactic-co-glycolic acid) (PLGA) copolymer is widely used due to its biocompatibility, biodegradability, and capacity to encapsulate drugs with varying solubilities under controlled release.^45^ Owing to its physicochemical properties, particularly its hydrophobic backbone and tunable glass transition temperature, PLGA has been used to efficiently encapsulates a broad range of therapeutic agents, such as chemotherapeutics e.g., doxorubicin (DOX), paclitaxel (PTX), anti-inflammatory compounds, and photosensitizers,^46,47^ PLGA-based NPs have been functionalized with PMs, combining efficient drug delivery with biomimetic targeting across diverse applications.^22,48,49^ Lipophilic drugs such as PTX are predominantly entrapped within PLGA NPs through hydrophobic and van der Waals interactions with the polymer matrix, while amphiphilic drugs such as DOX engage in additional ionic and hydrogen bonding with the carboxyl end groups of PLGA, enhancing encapsulation efficiency and stabilizing drug incorporation within the polymer network. The biodegradable nature of PLGA further enables controlled and sustained drug release, driven by polymer hydrolysis and diffusion of the encapsulated agents through the gradually eroding matrix.^50,51^ Lipid-based carriers, particularly liposomes, represent promising cores for PM coating due to their biocompatibility and adjustable composition.^52^ They provide high drug-loading efficiency and controlled release behavior, and when used to encapsulate chemotherapeutics such as DOX or PTX, have demonstrated superior therapeutic performance and attenuated off-target toxicity in preclinical cancer models.^53,54^

Inorganic NPs, particularly mesoporous silica nanoparticles (MSNs) and superparamagnetic iron oxide nanoparticles (SPIONs), have been increasingly explored as core materials for PM coatings.^24,30,55,56^ These nanocores are highly versatile, as their physicochemical properties can be precisely tuned to achieve multifunctionality, controlled drug release, and excellent biocompatibility. The ordered mesoporous framework of MSNs offers a large internal surface area (up to 1000 m^2^/g) and adjustable pore diameters (2–50 nm), allowing efficient loading and delivery of hydrophilic/hydrophobic therapeutic agents.^57,58^ These materials provide high drug-loading capacity, tunable pore structure, and additional therapeutic functionalities. Studies have demonstrated that hydrophobic surface modification of mesopores, such as octyl functionalization, enhances the entrapment and sustained release of hydrophobic drugs, such as docetaxel (DTX). This tunable mesoporous architecture has also been leveraged to co-encapsulate DOX and photosensitizers, enabling synergistic chemo-photothermal or photodynamic therapy (PDT).^7,43,59^

In contrast, superparamagnetic iron oxide nanoparticles (SPIONs) provide distinct advantages through their magnetic and photothermal properties, enabling multimodal applications such as magnetic resonance imaging (MRI), magnetic hyperthermia, and magnetically guided drug delivery. Its surface modification with polymers or silane-based ligands facilitates drug loading through electrostatic and coordination interactions. Li *et al.* illustrated that PM-functionalized, L-arginine-loaded magnetic NPs enable targeted nitric oxide (NO) release, promoting vascular repair and neuroprotection in ischemic stroke.^31^ Despite the availability of FDA-approved NP cores such as PLGA,^60^ no membrane-coated systems have reached clinical translation. Persistent challenges include variability in coating efficiency, incomplete preservation of functional membrane proteins, and potential immunogenicity.^16^ These limitations highlight a recurring theme across EV-mimetic systems: the biological interface between natural membranes and synthetic cores must be engineered with precision to preserve key membrane functionalities while ensuring manufacturability and regulatory compatibility. To advance the field, scalability should be considered from the earliest stages of NP design, ensuring that both NP synthesis and membrane coating are reproducible at clinically relevant volumes.

A critical but often overlooked issue behind these challenges is how the membrane physically wraps around the NP. This depends on the balance between the bending energy of the lipid bilayer and the adhesion energy between the NP and the membrane. This balance is also affected by the NP curvature and core stiffness, which, in turn, influence coating efficiency and stability. Complete wrapping occurs only when the adhesion energy is high enough to overcome the energy needed to deform the membrane. Therefore, changes in NP size, curvature, or mechanical properties can lead to incomplete coating, poor structural stability, or loss of functional membrane proteins. These effects likely contribute to the variability in coating efficiency seen across different systems.^61,62^ A better understanding of membrane wrapping, together with rational core selection, robust engineering, and standardized production strategies, is essential to improve coating uniformity, preserve functional membrane proteins, and move membrane-coated nanocarriers closer to clinical and regulatory-compliance.

## V. QUALITY CONTROL AND CHARACTERIZATION OF PM–NPS

Several analytical methods are used to evaluate PMs and PM–NPs, but each provides only partial information (Table I).^63^ A robust assessment, therefore, requires a combination of orthogonal techniques.

**TABLE I.** Common analytical methods and their main limitations in PM–NP characterization.

Method	Purpose	Main limitation
DLS and ELS	Measures particle size/PDI and zeta potential.	Sensitive to aggregation; cannot distinguish coated from uncoated NPs.
TEM/cryo-EM/FESEM	Visualizes morphology and core–shell structures.	Sample preparation may deform membranes; dense inorganic cores can obscure coatings.
Western blot (WB)	Detects platelet-specific membrane protein expressed on NPs.	Bulk measurement; cannot distinguish free membranes/p-EVs from NP-bound membranes.
NTA	Measures size distribution and particle concentration.	Cannot differentiate PM–NPs from free platelet vesicles or membrane debris.
Conventional flow cytometry	Measures platelet surface marker expression.	Detection threshold ≥140 nm; limited accuracy for small NPs, and cannot resolve single vesicles.
Nano-flow cytometry (e.g., NanoFCM, CytoFLEX Nano)	Single-particle phenotyping of PM–NPs and platelet EVs; quantifies surface markers (e.g., CD41, CD61, CD62P, and CD47); differentiates coated NPs from free vesicles.	Requires careful calibration and fluorescence optimization; limited fluorophore panel compared with conventional cytometers; potential swarm detection at high concentrations.

**Physicochemical characterization.** Dynamic light scattering (DLS) is commonly used to assess changes in hydrodynamic size, whereas electrophoretic light scattering (ELS) is used to determine zeta potential, both before and after membrane coating.^64^ While useful, DLS is highly sensitive to aggregation, and when used to assess a mixture of PM, NP, and PM–NP, it cannot reliably distinguish coated from uncoated nanoparticles. Microscopic techniques, including transmission electron microscopy (TEM), cryogenic electron microscopy (cryo-EM), and field emission scanning electron microscope (FESEM), can visualize core–shell structures, but fragile membranes may collapse during staining or become difficult to detect when NP cores are highly electron-dense.^65,66^

**Biochemical control of membrane components.** Western blotting (WB) can verify the presence of platelet-specific proteins on PM–NPs, but it cannot differentiate proteins belonging to intact coated NPs from those associated with contaminating free membranes.^67–69^

**Nanoparticle tracking analysis (NTA)** provides complementary information on particle size distribution and concentration, offering better resolution of polydispersity (PDI) than DLS. However, NTA cannot distinguish between free platelet-derived extracellular vesicles (p-EVs), membrane fragments, and coated NPs and is influenced by optical sensitivity and operator variation.^70,71^

**Surface marker analysis.** Flow cytometry enables quantitative detection of platelet proteins such as CD41, CD47, and P-selectin on PM–NP surfaces. It provides high-throughput analysis but is limited by detection thresholds of ∼140 nm (instrument-dependent). Smaller nanocarriers often require signal amplification strategies or bead coupling.^72^ Recent advances in multiparametric and nano-scaled flow cytometry now enable true single-vesicle phenotyping of NPs and EVs in the 40–200 nm range.^73^ By integrating ultrasensitive scatter detection with high-dimensional fluorescence profiling, these platforms can resolve nanoscale subpopulations based on both surface markers and membrane biophysical features, providing quantitative readouts of marker abundance per particle (e.g., CD41, CD61, CD62P, CD47) with substantially higher fidelity than conventional flow cytometry. Single-vesicle cytometry approaches, as exemplified by recent multiparametric EV flow cytometry and high-content platelet cytometry,^74^ offer sufficient resolution to discriminate engineered NPs from native platelet-derived vesicles, capture rare or dynamic subpopulations, and minimize swarm artifacts even in complex or heterogeneous samples. These capabilities enable robust statistical assessment of coating heterogeneity, particle identity and purity, and biologically driven population remodeling, providing powerful analytical advantages for the rigorous characterization and quality control of PM- and p-EV-coated nanocarriers.

**Need for separation methods and orthogonal validation.** Because most analytical tools cannot inherently discriminate coated from uncoated particles, effective separation of free membranes (e.g., density gradients, asymmetric-flow field-flow fractionation, SEC) is essential before characterization. A reliable QC panel can therefore rely on complementary physicochemical, biochemical, and functional assays capable of demonstrate coating efficiency, stability, and batch-to-batch reproducibility.

These limitations highlight the need for complementary analytic tools and strategies as well as effective methods to separate free membranes from PM-coated NPs. A robust QC framework combining orthogonal methods is therefore required to provide conclusive evidence of PM–NP formation, coating efficiency, and stability.

As biomimetic nanomedicine gradually moves closer toward clinical use, the selection of characterization tools must balance analytical resolution with industrial speed and scalability. Cryo-EM offers clear structural proof of a core–shell architecture, but it is time-consuming, expensive, and requires complex sample preparation, making it unsuitable for routine industrial QC. For large-scale manufacturing, DLS remains widely used because it is fast, scalable, and can potentially be integrated inline through spatially resolved DLS (SR-DLS), enabling real-time monitoring of particle size during assembly. However, DLS provides only average size data for the whole particle population and does not provide information on particle concentration and surface phenotype.^75^ To address these limitations, nano-flow cytometry (nFCM) has emerged as a high-throughput technique that can analyze thousands of particles per minute and measure particle size, concentration, and surface protein expression at the single-particle level. Compared with NTA, which typically detects particles down to ∼70 to 90 nm, nFCM can detect and characterize individual vesicles and NPs down to ∼40 nm. This provides better resolution for small EVs and membrane-coated NPs. The use of these high-throughput and automated methods will be essential to ensure batch-to-batch reproducibility and the level of process control needed for GMP compliance.^76,77^

## VI. CONFIGURATIONS OF PM-COATED NPS

### A. Single-membrane platelet-coated nanoparticles

One of the most straightforward strategies is to apply a single PM layer around NP cores, creating a natural core–shell structure. Wu *et al.* demonstrated this approach by coating DOX-loaded polypyrrole (PPy) NPs with PMs, resulting in targeted delivery and combined chemo-photothermal therapy (PTT) that achieved synergistic antitumor effects in an orthotopic nude mouse model of hepatocellular carcinoma (HCC).^78^ Other preclinical studies confirm the versatility of this design (Table II). PM-coated PLGA NPs, when loaded with PTX, have been shown to overcome multidrug resistance in breast cancer,^47^ decrease inflammatory responses in acute lung injury,^22^ and promote angiogenesis in ischemic tissue.^48^ These examples highlight the adaptability of single-membrane PM–NPs across diverse preclinical disease models. Because cancer patients face a risk of venous thromboembolism (VTE), with 20%–30% of cases linked to tumor presence, strategies that exploit the natural thrombus-homing ability of platelets are particularly relevant. This risk is driven by surgery-related vascular injury, tumor-derived procoagulant proteins, and chemotherapy-induced vascular dysfunction. Systemic administration of nanosystems may further increase thrombotic risks.^79^ To address this, platelet-inspired nanocarriers have been engineered as targeted carriers for thrombolytic therapy, aiming to improve clot penetration and dissolution while minimizing systemic bleeding. Wang *et al.* reported that platelet-cloaked silica NPs enhanced thrombus retention, enabling precise arteriovenous thrombolysis. In another approach, PM-coated silica NPs targeted damaged vasculature, permitting the co-delivery of vascular-disrupting and anti-angiogenic agents for simultaneous vessel disruption and thrombolysis.^24,30^ In these single-membrane systems, the NP core acts as a drug reservoir and provides additional therapeutic functions, while the PM ensures immune evasion, extended circulation, and selective targeting. To achieve these effects reproducibly, quantitative control of membrane coverage, protein orientation, and the PM:NP ratio is critical, as even subtle differences in coating uniformity can influence biodistribution, opsonization, and thrombus- or tumor-homing efficiency. Nevertheless, achieving reproducible performance requires careful optimization of the PM/NP ratio. Insufficient membrane coverage leads to incomplete coating, instability, and reduced function.^17^ Robust preclinical evaluation and translational studies remain essential to advance these multifunctional PM–NPs toward clinical application.

**TABLE II.** Selection of preclinical studies investigating the use of single platelet membrane-coated nanoparticles (PM–NPs).

	Core material	Therapeutic modality	Therapeutic release triggers	Disease model/application	Key therapeutic outcomes	References
PM-coated polymeric NPs	HGF-PLGA	Therapeutic angiogenesis	Hypoxic ischemic microenvironment	Hindlimb ischemia (in vitro and in vivo, mouse)	Restored tissue perfusion	48
	PTX-PLGA	Chemotherapy	Acidic pH (TME)	Multidrug-resistant breast cancer (MDR)-(in vitro and in vivo, mouse)	Enhanced tumor accumulation and cytotoxicity; overcame MDR	47
	ICG-PLGA	Photothermal and radiotherapy	X-ray irradiation and NIR activation	4T1 breast cancer cells (in vitro and in vivo, mouse)	Improved tumor accumulation and therapeutic efficacy	80
PM-coated inorganic nanoparticles	Hirudin-MnOx@Ag2S	Antithrombotic, photothermal, and chemodynamic therapy	Thrombin (hirudin binding), NIR-II irradiation (Ag₂S PTT), GSH (MnOx decomposition → Mn²⁺ → •OH)	4T1 breast cancer cells (in vitro and in vivo, mouse)	Simultaneous thrombus elimination and tumor suppression	79
	MSN@PM-C-A	Intratumoral vascular disrupting and anti-angiogenesis	CA4-induced microthrombosis enables sustained Apa release	MHCC-97H hepatocellular carcinoma (in vitro and in vivo, mouse)	Synergistic vessel impairment, shutdown of tumor blood supply, enhanced tumor suppression	30

### B. Platelet-hybrid membrane coated nanoparticles

Hybrid membrane-coated NPs are emerging as versatile platforms for drug delivery, detoxification, cancer diagnosis, and vaccination. Unlike single-membrane systems, these particles are fabricated by fusing two or more natural cell membranes, such as PMs with tumor, leukocyte, or erythrocyte membranes, and coating them onto NP cores.^81^ This approach integrates complementary biological traits into a single platform, extending capabilities beyond those of individual membranes.^82–84^ The intrinsic features of PMs, including CD47-mediated immune evasion and GPIbα-driven vascular adhesion, provide a strong foundation.^34^ Hybridization adds further advantages. For example, combining PMs with cancer cell membranes (CCMs) bearing tumor-associated antigens (TAAs) enables NPs to benefit from both platelet-mediated vascular targeting and tumor homotypic recognition. Li *et al.* demonstrated this concept with platelet-cancer cell hybrid membrane-coated lipid NPs co-loaded with sorafenib and triptolide, which achieved precise dual targeting and improved therapeutic efficacy in HCC preclinical mouse models.^85^ Similarly, Lingling *et al.* used platelet-tumor hybrids for preclinical glioma therapy, reporting superior drug accumulation and antitumor outcomes.^49^ Leukocyte membranes have also been integrated to exploit their roles in immune surveillance. Adhesion molecules such as lymphocyte function-associated antigen 1 (LFA-1), which binds intercellular adhesion molecule 1 (ICAM-1) on tumor vasculature, and PSGL-1, which interacts with platelet P-selectin, enable cooperative trafficking and tumor infiltration.^86^ Zhang *et al.* developed MSN NPs cloaked with platelet-leukocyte hybrid membranes, co-loaded with both chemo- and phototherapy agents. This design facilitated vascular rolling, tumor penetration, and accumulation in triple-negative breast cancer (TNBC), producing markedly improved outcomes compared with single-membrane systems in a TNBC mouse preclinical model.^59^ Overall, platelet-hybrid systems combine the drug-loading and tunable properties of synthetic cores with synergistic biological functions, offering greater precision and efficacy than single-membrane coating platforms, as shown in Table III.

**TABLE III.** Selected studies investigating hybrid platelet membrane-coated nanoparticles (PM–NPs) and their applications in preclinical disease models. Abbreviations: platelet membrane (PM), cancer cell membrane (CCM), leukocyte membrane (LM), poly (lactic-co-glycolic acid) (PLGA), mesoporous silica nanoparticle (MSN), doxorubicin (DOX), and nanoparticle (NP).

Hybrid system	Main functions	Core material/formulation	Therapeutic modality	Disease application	References
Platelet-cancer cell hybrid	PM: immune evasion CCM: homologous tumor targeting	β-mangostin-PLGA nanoparticles	Chemotherapy	Glioma (*in vitro* and *in vivo*, mouse)	49
Platelet-cancer cell hybrid	PM: immune evasion and prolonged circulation CCM: homologous tumor targeting	Sorafenib/triptolide lipid nanoparticles	Dual-drug chemotherapy	Hepatocellular carcinoma (HCC) (*in vitro* and *in vivo*, mouse)	85
Platelet-leukocyte hybrid	PM: immune evasion and tumor targeting LM: vascular rolling; adhesion; tumor infiltration	IR780/DOX-loaded-MSNs	Chemotherapy, photothermal, photodynamic therapy	Triple-negative breast cancer (TNBC) (*in vitro* and *in vivo*, mouse)	59
Platelet-erythrocyte hybrid	PM: vascular targeting EM: prolonged circulation; immune evasion	JQ1-loaded PLGA NPs	Targeted delivery, controlled release	Cardiovascular disease (*in vitro* and *in vivo*, mouse)	23

Nonetheless, major engineering and translational challenges remain. Standardized protocols for hybrid membrane fusion are lacking, coating quality and reproducibility are uncertain, and the need to harmonize storage conditions for different membrane types complicates scale-up. Moreover, robust assays to quantitatively confirm hybridization and monitor therapeutic payload release are still limited. In addition, distinguishing true membrane fusion from simple co-vesiculation or physical mixing remains analytically challenging, underscoring the need for single-vesicle assays capable of resolving hybrid membrane topology, fusion efficiency, and relative membrane contribution. At present, platelet-hybrid NPs remain at the proof-of-concept stage, and their progression toward large-scale manufacturing and regulatory approval will require systematic validation and rigorous QC.

## VII. PLATELET-DERIVED EXTRACELLULAR VESICLES (P-EVS) AS A COATING SOURCE FOR NPS

p-EVs represent a heterogeneous population of microvesicles and exosomes released from platelets through activation, shear, or storage lesions, and are the abundant circulating EVs in blood.^21,25,87^ They carry a subset of PM proteins and cytosolic components. In addition to common EV markers such as CD9, CD63, TSG101, and ALIX,^88^ p-EV membranes retain platelet-specific proteins including PECAM-1 (CD31), GPIIb/IIIa (CD41/CD61), GPIbα (CD42b), P-selectin (CD62P), and phosphatidylserine (PS).^89,90^ These markers reflect their platelet origin and allow p-EVs to preserve several biological functions of platelets, while exerting active roles in disease biology. Their interactions with vascular and tumor-associated ligands support targeting, while their nanoscale size improves circulation, tissue penetration, and biological stability.^21,91^ p-EVs also express proteins such as CD46 and CD63, which may facilitate transport across the blood–brain and blood–retinal barriers.^92^ Their vesicular structure tolerates freezing, thawing, and lyophilization better than bulk PM preparations, especially with appropriate cryoprotectants.

PCs are an abundant and practical source of p-EVs. A standard 200-ml platelet concentrate contains 4–7 × 10^11^ platelets and typically yields 10^13^–10^14^ p-EVs, based on estimates of 10^11^–10^12^ p-EVs/ml of supernatant.^93^ However, collection method, leukoreduction, pathogen-reduction treatments, and the use of platelet additive solutions may affect p-EV yield and composition.^94–96^ p-EVs can be isolated using scalable and translationally compatible methods such as size-exclusion chromatography (SEC), ultracentrifugation (UC), or tangential flow filtration.^21,93^ The isolation method plays a critical role because it affects vesicle purity, surface composition, and protein corona formation. These factors directly influence the biological activity and coating performance of p-EVs. UC is widely used because it is accessible and can process relatively large volumes, but it may cause vesicle aggregation and co-isolation of plasma proteins and lipoproteins. These contaminants can form a protein corona that may mask important platelet ligands, such as GPIbα, which is involved in thrombus targeting, and CD47, which contributes to “don't eat me” signaling. This may reduce how well the coating mimics the native PM. By contrast, the SEC separates vesicles from soluble plasma proteins based on hydrodynamic size. This generally results in purer vesicle fractions and better preserves the native membrane surface.^97^ As emphasized in the MISEV guidelines, isolate purity should be assessed using measures such as protein-to-particle ratios. This is important to ensure that the biological activity of membrane-coated NPs comes from the membrane itself and not from co-isolated plasma contaminants.^76^

Given their molecular composition, biological stability, and availability from existing blood bank workflows, p-EVs provide a promising coating source for NPs. Their surface markers suggest potential to confer immune evasion, vascular adhesion, and tumor-homing capabilities when applied as cloaking layers. These features mirror the broader landscape of engineered EV-based therapeutics, where donor cell selection, EV biogenesis, and downstream processing are integrated into an end-to-end manufacturing scheme.^98^

## VIII. THERAPEUTIC APPLICATIONS

### A. p-EV-NPs vs PM–NPs

p-EVs and PMs are both platelet-derived but differ somewhat in structure, production methods, and translational suitability. PMs rely on the full set of platelet surface receptors, such as CD47, GPIbα, integrins, and CD62P, to support immune evasion and vascular adhesion as indicated above.^13^ However, PM preparation requires platelet lysis, membrane purification, and vesiculation, processes that increase variability and predispose the membranes to aggregation or protein denaturation during freeze–thaw cycles.^22^ Their larger hydrodynamic size also contributes to faster clearance from circulation compared with p-EVs. p-EVs are naturally secreted vesicles that maintain platelet identity while benefiting from smaller size, intrinsic bioactive cargo, and greater physical stability. Their expression of CD46 and CD63 supports potential barrier-crossing functions.^92^ From a manufacturing perspective, p-EVs are simpler to obtain: they are collected directly from PC supernatants and isolated using well-established methods as described above.^99^ Their nanoscale structure is more compatible with frozen or lyophilized storage, enabling more robust handling compared with PMs.^21^ Together, these differences suggest that while both PMs and p-EVs offer biological advantages for NP coatings, p-EVs may provide improved stability, scalability, and regulatory adaptability. PMs remain valuable when full platelet-receptor complexity is required, particularly for vascular or thrombus-targeted applications, but p-EVs represent an increasingly attractive platform for clinical translation. Notably, non-platelet EV systems have already demonstrated how exocytosed, exosome-cloaked inorganic nanocarriers can achieve enhanced tissue targeting and disease modulation *in vivo*, providing a conceptual blueprint for future platelet-EV-based artificial exosome designs.^100^

Having outlined the main platelet-derived coating materials (PMs and p-EVs) and their relative advantages, we next review how PM–NPs have been applied in preclinical disease models. PM–NPs have emerged as adaptable therapeutic platforms across cancer, cardiovascular disorders, and infectious diseases.^10,66^ This versatility is driven by their capacity to load a wide range of therapeutic cargos, including chemotherapeutic drugs, photosensitizers for phototherapy, nucleic acids for gene modulation, and immunomodulators for inflammatory control. Their intrinsic targeting ability, combined with immune evasion and controlled drug release, enables multifunctional treatment strategies. Representative examples are summarized in Tables II and III.

### B. PM–NPs in cancer therapy

**FIG. 3.**
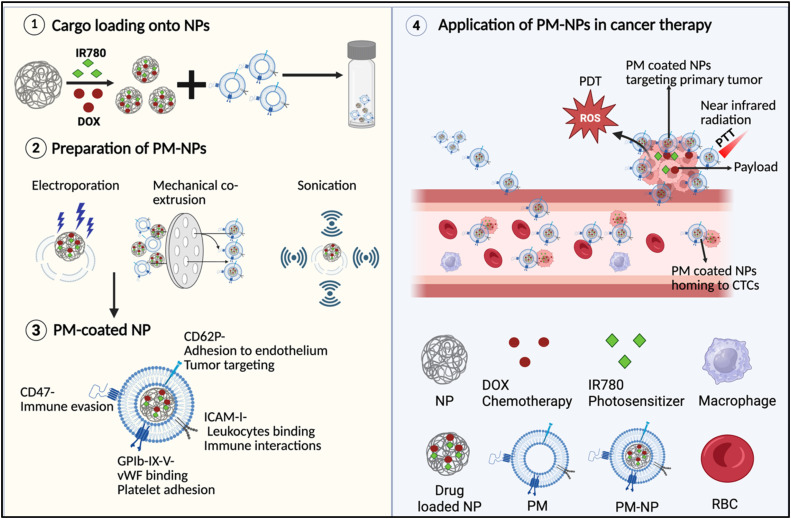
Schematic illustration of the preparation and targeted therapeutic application of platelet membrane-coated nanoparticles (PM–NPs). (1) Loading of therapeutic cargos onto NP cores. (2) Preparation of PM–NPs. (3) Structure and targeting mechanism. (4) Application in cancer therapy. Abbreviations: platelet membrane (PM), nanoparticle (NP), platelet membrane-coated nanoparticle (PM–NP), doxorubicin (DOX), photosensitizer (IR780), reactive oxygen species (ROS), photothermal therapy (PTT), photodynamic therapy (PDT), circulating tumor cell (CTC), and red blood cell (RBC).

Platelets naturally accumulate at tumor sites through receptor–ligand interactions. Surface markers such as P-selectin (CD62P) and glycoproteins GPIb and GPIIb/IIIa bind to ligands including CD44, von Willebrand factor (vWF), and fibrinogen, all of which are abundant in the tumor microenvironment. By leveraging these interactions, PM–NPs can achieve efficient tumor homing and targeted drug delivery (Fig. 3).^35,101^ Systemic toxicity and multidrug resistance remain major challenges in chemotherapy, largely due to the nonspecific distribution of free drugs. PM–NPs may address such limitations by being targeted carriers for chemotherapeutic agents. In addition, PMs express CD47, an immune checkpoint protein that interacts with SIRPα on macrophages and delivers a “don't-eat-me” signal, thereby prolonging circulation and enhancing tumor accumulation.^102^ The synthetic NP core enables high drug loading and tunable release kinetics, supporting controlled and sustained delivery. PM–NPs have also been developed for combination therapies. Ye *et al.* engineered bio-inspired nanoplatelets for chemo-photothermal therapy, which effectively inhibited breast cancer metastasis.^103^ In another study, exosome-coated porous silica nanocarriers derived from tumor cells have demonstrated that EV cloaking can enhance intratumoral retention, cancer stem cell targeting, and overall chemotherapy efficacy.^104^ Although these systems do not employ platelet sources, they highlight how combining a drug–reservoir inorganic core with biologically specific vesicle coatings can expand the therapeutic window—an approach that could be translated to platelet- and p-EV-based platforms. Together, these findings highlight the potential of PM–NPs to deliver targeted, multifunctional cancer therapy that reduces systemic toxicity, overcomes drug resistance and improves therapeutic efficacy.

### C. PM–NPs in cardiovascular disease

Cardiovascular diseases (CVDs) are the leading cause of morbidity and mortality worldwide, accounting for an estimated 17.8 × 10^6^ deaths in 2017.^10^ Despite existing treatments, challenges such as poor drug targeting, systemic side effects, and limited therapeutic efficacy persist. PMs offer a natural solution due to their phospholipid bilayer, enriched with receptors, glycoproteins, and integrins that mediate adhesion to damaged or inflamed vasculature. Platelets recognize vascular injury through GPIb, which binds exposed von Willebrand factor (vWF), and through integrin αIIbβ3, which interacts with fibrinogen and activated endothelial cells.^105^ These interactions enable PM-coated NPs to localize selectively to sites of vascular damage or thrombosis, making platelet membranes an attractive platform for biomimetic drug delivery in cardiovascular medicine.^105^ Once localized, the therapeutic effect is driven by the NP core, which releases its payload in a controlled and targeted manner. *Li *et al.** engineered platelet membrane-coated magnetic nanoparticles (PAMNs) loaded with L-arginine. Under guidance from an external magnetic field, these nanocarriers accumulated at ischemic stroke lesions and generated nitric oxide (NO) *in situ*, promoting vasodilation and reperfusion in early stroke mouse models.^31^ In another study, platelet membrane-coated PLGA microbubbles demonstrated potential for noninvasive ultrasound imaging of myocardial ischemia–reperfusion (MI/R) injury. These PM-coated microbubbles showed enhanced adhesion to damaged endothelium compared with uncoated controls, enabling more sensitive detection of early cardiac injury.^106^ Together, these findings highlight the promise of PM–NPs as targeted therapeutic and diagnostic platforms for CVDs. Their intrinsic vascular affinity improves localization and may enhance efficacy, while reducing off-target effects compared with conventional treatments.

### D. PM–NPs in infectious diseases

Platelets naturally interact with a broad range of pathogens, including viruses, bacteria, and parasites, making PM–NPs promising carriers for antimicrobial agents. Conventional antibiotics often suffer from poor specificity, systemic toxicity, and the rapid emergence of resistance.^12^ PM–NPs offer a biomimetic strategy to overcome these limitations by leveraging the inherent affinity of platelets for pathogens and inflamed tissues. A key mechanism is toxin binding. Platelets express ADAM10, a receptor for *Staphylococcus aureus* α-toxin, which contributes to their capacity to bind bacterial virulence factors.^107^ Since *S. aureus*, particularly methicillin-resistant strains (MRSA), induces immune dysregulation through secreted toxins such as α-toxin, toxin neutralization has emerged as a therapeutic approach. PM–NPs can function as natural decoys, sequestering bacterial toxins to prevent cytolysis while simultaneously delivering antimicrobial payloads. Another mechanism involves pathogen binding and interference, where PM–NPs directly adhere to circulating microbes, blocking their ability to interact with host cells.^66^ CD47 expression further allows PM–NPs to evade phagocytic clearance, prolonging circulation and enhancing antimicrobial delivery. Hu *et al.* engineered biomimetic PM–NPs that neutralized *S. aureus* α-toxin, protecting against lethal systemic infection and demonstrating strong therapeutic potential.^108^ Hybrid membrane designs have also been applied. An erythrocyte–platelet hybrid membrane nanoplatform containing Fe_3_O_4_ and cinnamaldehyde was developed for lung-targeted antibacterial therapy. Under ultrasonic stimulation, these particles induced ferroptosis in MRSA by elevating reactive oxidative species (ROS) levels and promoting lipid peroxidation, significantly reducing bacterial burden and improving survival in a mouse pneumonia model.^109^ These studies illustrate the promise of PM–NPs as multifunctional tools for infectious disease therapy, combining toxin neutralization, pathogen binding, and controlled antimicrobial release.

## IX. KEY TRANSLATIONAL CHALLENGES AND OUTSTANDING QUESTIONS

Despite encouraging progress, the clinical translation of PM- and p-EV-based nanocarriers keeps facing several unresolved challenges (see Subsec. IX A).^67^ A primary issue is reproducibility and standardization. It remains clear that the protocols used for membrane isolation, vesicle preparation, coating, as well as QC vary widely among laboratories. Current coating approaches, including extrusion, sonication, and microfluidics, often yield inconsistent results, leading to partially coated NPs and substantial batch-to-batch variability.^17^ A second challenge is GMP-compliant membrane sourcing. Platelets are biological materials subject to donor-to-donor variability, activation state changes, and storage-dependent alterations in membrane composition. Ensuring safety of both PMs and p-EVs requires rigorous pathogen testing and reduction treatments, which adds complexity. PM preparation requires destructive lysis and multistep purification that increase variability and contamination risks. Although a few NP-based therapies have entered clinical trials, no PM- or p-EV-coated nanocarrier has yet progressed to clinical evaluation. A further challenge is the lack of a clearly defined product specification for PM- and p-EV-coated nanocarriers. As hybrid constructs positioned within the broader EV-based therapeutic continuum, they do not fit neatly into existing categories for biologics, EVs, or synthetic nanomedicines, making it essential to define critical quality attributes (CQAs) such as coating completeness, membrane orientation, marker density, and PM:NP ratios in a way that is compatible with regulatory expectations. Immunogenicity is another consideration for clinical translation. PMs contain HLA, ABO blood group antigens, and platelet-specific antigens (HPA), which are known to induce alloimmune responses in transfusion settings, especially after repeated exposure, but can be limited using the immunohematology testing methods developed in transfusion medicine. Aggregation or protein denaturation during membrane processing can further increase complement activation. p-EVs likely present a lower immunogenic risk because of their smaller size, reduced antigen load, and their natural presence in circulation, but high-dose or chronic administration has not been systematically evaluated. Expertise from transfusion medicine, including antigen matching, pathogen reduction, and donor screening, can help reduce immunogenic risks and guide the development of safe, clinically compatible PM- and p-EV-based nanocarriers. No established assays currently exist to quantify the antigen load or immunogenic potential of PM-coated NPs, representing a major unmet need for regulatory evaluation. Emerging single-particle analytical platforms, such as nFCM and high-content platelet cytometry, may eventually enable more quantitative assessment of antigen density and heterogeneity at the vesicle or NP level, but their use in formal immunogenicity testing is still at an early stage. Overall, formal immunogenicity and safety studies remain essential, as no long-term human data are yet available. Storage stability is another major hurdle. PM vesicles readily aggregate and experience protein degradation during freeze–thaw cycles. p-EVs are more stable but still require optimized cryoprotectants and validated lyophilization protocols.^110^ The absence of standardized shelf-life studies limits reproducibility and regulatory readiness. Finally, scalable manufacturing and regulatory uncertainty represent substantial barriers. Translation will require scalable, GMP-compatible methods capable of producing uniform nanocarriers while preserving membrane integrity and functional protein orientation. Although EV-based therapeutics are beginning to enter regulatory frameworks, PM–NPs remain poorly defined within current guidelines. Regulatory agencies such as the FDA and EMA are expected to require detailed molecular characterization, validated manufacturing pipelines, potency assays, and comprehensive biosafety data. In line with emerging EV-oriented frameworks (e.g., the MISEV guidelines), harmonizing EV-style identity and purity criteria with nanomedicine-focused chemistry, manufacturing, and controls (CMC) requirements will be critical to position PM- and p-EV-based systems within a coherent regulatory pathway. Coordinated efforts in standardization, safety assessment, and scalable manufacturing will be essential to advance platelet-derived nanocarriers toward clinical application.

### A. Outstanding questions

• How can platelet membranes and p-EVs be produced with consistent quality? Donor variability and storage changes remain major obstacles.
• What methods can reliably confirm complete and stable membrane coating of nanoparticles? Current assays do not clearly distinguish partially coated from fully coated particles.
• How to control the potential immune risks of repeated dosing? Long-term effects, antibody formation, and complement activation remain to be evaluated.
• How can PMs and p-EVs be stored without losing function? Optimal conditions for freezing, thawing, and lyophilization need to be defined.
• Can scalable, GMP-compatible manufacturing be achieved? It is unclear which production platforms best support large-scale, standardized coating.
• For which clinical applications do PMs or p-EVs offer clear advantages? Direct comparisons with existing targeting strategies are still limited.
• How should PM- and p-EV-based nanocarriers be classified within current regulatory frameworks for EV therapeutics and nanomedicines, and which EV-derived guidelines (e.g., MISEV) are most relevant for defining their identity, purity, and potency?

## X. CONCLUSIONS

Platelet-derived membranes and p-EVs offer powerful strategies to create biomimetic nanocarriers with improved circulation, immune evasion, and disease-specific targeting. Despite existing preclinical results, no PM- or p-EV-coated NP has yet entered clinical trials, largely due to inconsistent coating efficiency and the lack of standardized large-scale manufacturing. Progress will depend on quantitative assays that can confirm coating quality and reproducibility, and, possibly, scalable engineering methods such as microfluidic assembly supported by automated QC. In parallel, aligning PM- and p-EV-based nanocarriers with EV-inspired analytical standards (for example, MISEV-type criteria for vesicle identity and purity) and with existing FDA/EMA expectations for biologics and nanomedicines will help to clarify regulatory positioning. To date, no PM–NPs or exosome/EV product has received FDA approval, and platelet membrane-based nanocarriers remain preclinical; however, EV-based therapeutics are being evaluated in early-phase clinical trials, highlighting growing regulatory interest in this class of products.^19,111,112^ Expertise from transfusion medicine will also be important to strengthen sourcing, pathogen safety, and immunological risk mitigation.^25^ With continued research efforts in these areas, platelet-derived NPs have the potential to become a clinically viable class of biomimetic delivery systems and open the production and regulatory pathways for other membrane-coated NPs.

## Data Availability

Data sharing is not applicable to this article as no new data were created or analyzed in this study.
